# Angiotensin II receptor blockers and gastrointestinal adverse events of resembling sprue-like enteropathy: a systematic review

**DOI:** 10.1093/gastro/goz019

**Published:** 2019-06-01

**Authors:** Ayesha Kamal, Christopher Fain, Angela Park, Peiqi Wang, Eduardo Gonzalez-Velez, Daniel A Leffler, Susan M Hutfless

**Affiliations:** 1 Department of Medicine, Division of Gastroenterology, Johns Hopkins University, Baltimore, MD, USA; 2 Johns Hopkins, Department of Surgery and Surgical Sciences, Johns Hopkins University, Baltimore, MD, USA; 3 Division of Gastroenterology, Beth Israel Deaconess Medical Center, Boston, MA, USA; 4 Department of Epidemiology, Johns Hopkins Bloomberg School of Public Health, Baltimore, MD, USA

**Keywords:** angiotensin II receptor blockers, olmesartan, enteropathy, diarrhea

## Abstract

**Background:**

Olmesartan, an angiotensin II receptor blocker (ARB), is associated with gastrointestinal symptoms resembling sprue-like enteropathy. Some have proposed that enteropathy may be a class effect rather than olmesartan-specific. We performed a systematic review to identify literature of sprue-like enteropathy for all ARBs.

**Methods:**

Case reports, case series and comparative studies of ARBs were searched on PubMed and Embase databases through 21 November 2018 and then assessed.

**Results:**

A total of 82 case reports and case series as well as 5 comparative studies, including 248 cases, were selected and analysed. The ARBs listed in the case reports were olmesartan (233 users; 94.0%), telmisartan (5 users; 2.0%), irbesartan (4 users; 1.6%), valsartan (3 users; 1.2%), losartan (2 users; 0.8%) and eprosartan (1 user; 0.4%). The periods between ARB initiation and onset of symptoms ranged from 2 weeks to 13 years. Histologic results were reported in 218 cases, in which 201 cases (92.2%) were villous atrophy and 131 cases (60.1%) were intraepithelial lymphocytosis. Human leucocyte antigen (HLA) testing was performed in 147 patients, among whom 105 (71.4%) had HLA-DQ2 or HLA-DQ8 haplotypes. Celiac-associated antibodies were tested in 169 patients, among whom 167 (98.8%) showed negative results. Gluten exclusion from the diet failed to relieve symptoms of enteropathy in 127 (97.7%) of 130 patients with information. Complete remission of symptoms after discontinuation of ARB was reported in 233 (97.4%) of the 239 patients with information. Seven cases (2.8%) reported recurrence of symptoms after restarting olmesartan; rechallenge was not reported for the non-olmesartan ARBs. The retrospective studies conducted worldwide had inconsistent study designs (e.g. differences in periods of study and case definition) and findings.

**Conclusions:**

Although enteropathy is rare, clinicians should remain vigilant of this potential adverse event even years after medication initiation.

## Introduction

Angiotensin II receptor blockers (ARBs) are among the most commonly used blood-pressure-lowering drugs in the world [1, 2]. They are often prescribed as the first-line anti-hypertensive for patients with diabetes and renal disease [2]. ARBs were first approved by the Food and Drug Administration for the treatment of hypertension in 1995 [3]. Losartan was the first ARB approved followed by irbesartan (1997), candesartan (1998), telmisartan (1998), valsartan (2002), olmesartan (2002) and eprosartan (2006). ARBs lower blood pressure by competitively inhibiting angiotensin II receptors, resulting in reduced vasoconstriction and aldosterone and catecholamine secretion [4].

In 2012, Rubio-Tapia *et al.* [5] reported unexplained chronic diarrhea and weight loss and sprue-like biopsy findings in 22 hypertensive patients treated with olmesartan. These patients had negative celiac serologies and did not respond to a gluten-free diet. Discontinuation of olmesartan resulted in histologic recovery or improvement and remission of symptoms in these patients. After this case series, more case reports described sprue-like disease with olmesartan use [6–9]. The pathogenesis of mucosal damage caused by olmesartan is not clearly understood but is considered to be immune-mediated inflammation [10]. This immune-mediated damage is manifested as partial to severe (total) intestinal villous atrophy with more variable intraepithelial lymphocytosis, frequently increased sub-epithelial collagen and inflammation of lamina propria [10]. Similar histological findings together with sprue-like enteropathy were reported for the first time in a patient receiving telmisartan in 2014 [11], followed by 11 case reports of enteropathy after non-olmesartan ARB use [10–17]. These case reports raised the possibility that ARB-related enteropathy may be a class effect rather than an effect specific to olmesartan [13].

However, limited epidemiological studies compared enteropathy outcomes between olmesartan and other anti-hypertensive medications, including other ARBs and angiotensin-converting enzyme (ACE) inhibitors, and results have been mixed. However, limited epidemiological studies compared enteropathy outcomes between olmesartan and other anti-hypertensive medications, including other ARBs and ACE inhibitors, and results have been mixed. We performed a systematic review of the published literature to identify case reports, case series and comparative studies of ARB-related enteropathy.

## Methods

### Literature sources

We performed a literature search through 21 November 2018 in PubMed and Embase databases. We searched the articles using international ARB brand and generic drug names according to Micromedex/Martindale to have the broadest search possible [18, 19]. Micromedex is an online evidence-based database that includes referenced information about drugs and toxicology to health-care professionals [18]. Martindale database is published as a reference book annually containing information on drugs in clinical use worldwide [19]. The following search string was used: olmesartan or losartan or irbesartan or candesartan or telmisartan or valsartan or eprosartan and retrospective or prospective or database or case. The search did not include outcomes to capture all potential literature.

### Eligibility and study selection

Any article that mentioned an ARB including case reports, case series and comparative studies (prospective and retrospective) was included at the abstract level of review. Titles, abstracts and full texts were assessed independently by two reviewers (A.K. and A.P.) to determine the eligibility of the studies according to inclusion and exclusion criteria. If a case report did not mention diarrhea, it was excluded. All studies that did not include humans (i.e. animal studies, cell lines and spectrophotometry) were excluded. Comparative studies were included if they mentioned conditions associated with enteropathy, including celiac disease. During the full-text review, the reviews, editorials and guidelines were excluded. Studies that reported on conditions or symptoms consistent with enteropathy were included. Online systematic review management software, Covidence, was used for title/abstract screening, full-text screening, data abstraction and quality assessment [20]; it was designed by researchers familiar with the systematic review process in order to make conducting reviews more efficient [21].

### Data collection

Two reviewers (A.K. and A.P.) extracted demographic, exposure and outcome information from the included studies into Microsoft Excel 2013. The data were independently collected for each study and were cross-checked. Discrepancies were resolved through consensus. If multiple articles reported on the same patients, only the later report of the patient was included. We extracted the following information for case reports and case series: information on demographics; type of ARB used; the period between the ARB initiation and symptom onset; HLA-DQ haplotypes; antibodies associated with celiac disease; response to gluten exclusion from the diet; and symptomatic, endoscopic and histologic response to ARB discontinuation and response to resuming ARB use (dechallenge/rechallenge). For the comparative studies, we extracted information on demographics, outcome definition and rate of intestinal malabsorption among olmesartan, other ARBs and angiotensin-converting-enzyme-inhibitor (ACEI) users. We collected information on the factors adjusted for the analysis and required that the extracted rate adjusted for a minimum of age and sex.

## Results

### Characteristics of included studies

**Figure 1.**
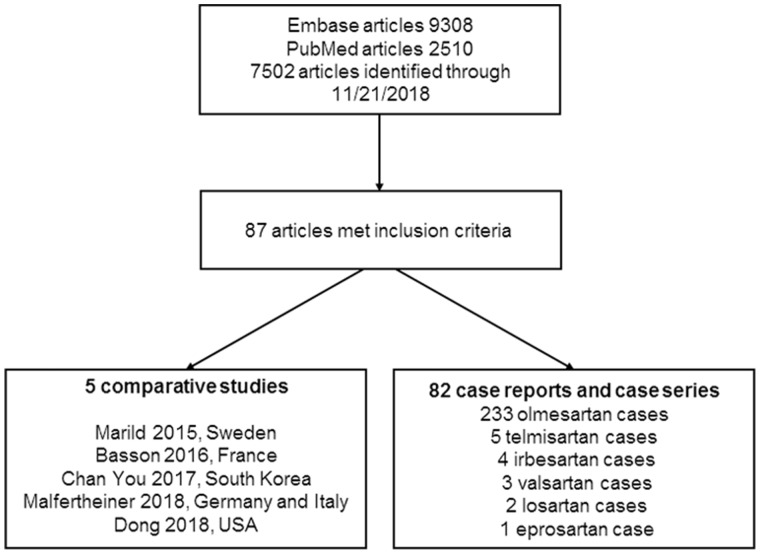
Study selection process

The final search yielded 2510 articles in PubMed and 9308 in Embase before de-duplication. The total number of articles screened at the title/abstract level was 7502 articles; 82 articles reporting cases and 5 comparative studies were included. These studies were published between 2012 and 2018 (Figure 1).

### Case reports and case series

#### Characteristics of patients

There were 248 cases resembling sprue-like enteropathy (age at diagnosis 45–89 years; 57.3% female) (Table 1). The ARBs listed in the case reports were olmesartan (233 cases; 94.0%), telmisartan (5 cases; 2.0%) [11–14], irbesartan (4 cases; 1.6%) [13, 16, 22], valsartan (3 cases; 1.2%) [13, 15], losartan (2 cases; 0.8%) and eprosartan (1 case; 0.4%) [17]. The periods between ARB initiation and onset of symptoms ranged from 2 weeks to 13 years [23–25].

**Table 1.** Summary of 248 patients with angiotensin II receptor blocker (ARB)-related enteropathy

Distribution of cases	
Olmesartan	233 (94.0%)
Telmisartan	5 (2.0%)
Irbesartan	4 (1.6%)
Valsartan	3 (1.2%)
Losartan	2 (0.8%)
Eprosartan	1 (0.4%)
Age range at diagnosis	45–89 years
Female	57.3%
ARB use to symptoms	2 weeks to 13 years
Histologic results	
Villous atrophy	192/204 olmesartan 4/4 telmisartan 2/4 irbesartan 2/3 valsartan 0/2 losartan 1/1 eprosartan
Intraepithelial lymphocytosis	126/204 olmesartan 1/4 telmisartan 2/4 irbesartan 2/3 valsartan 0/2 losartan 0/1 eprosartan
HLA-DQ2 or HLA-DQ8 haplotypes	105/145 olmesartan 0/2 irbesartan
Remission of symptoms after discontinuation of ARB	218/224 olmesartan 5/5 telmisartan 4/4 irbesartan 3/3 valsartan 2/2 losartan 1/1 eprosartan

HLA, human leucocyte antigen.

#### Laboratory evaluation

Among 147 patients who underwent HLA testing, 105 (71.4%) presented HLA-DQ2 or HLA-DQ8 haplotypes (105/145 olmesartan users, 0/2 irbesartan users). Among 169 patients in whom celiac-associated antibodies were tested, 167 (98.8%) showed negative results (157/159 olmesartan users, 4/4 telmisartan users, 4/4 irbesartan users, 1/1 valsartan users, 1/1 eprosartan users); 2 (1.2%) olmesartan users showed positive results.

#### Histopathological findings

Histologic results were reported in 218 patients, among whom 201 (92.2%) had villous atrophy (192/204 olmesartan users, 4/4 telmisartan users, 2/4 irbesartan users, 2/3 valsartan users, 0/2 losartan users, 1/1 eprosartan users) and 131 (60.1%) developed intraepithelial lymphocytosis (126/204 olmesartan users, 1/4 telmisartan users, 2/4 irbesartan users, 2/3 valsartan users, 0/2 losartan users, 0/1 eprosartan users).

#### Clinical and histopathological outcomes

Gluten exclusion from the diet failed to resolve symptoms in 127 (97.7%) of 130 patients with information. Three olmesartan users responded to dietary exclusion; however, the dietary exclusion occurred while the olmesartan treatment was finished, and therefore exclusive response to gluten exclusion could not be established. Complete remission of symptoms after discontinuation of ARB was reported in 233 (97.4%) of the 239 patients with information (218/224 olmesartan users, 5/5 telmisartan users, 4/4 irbesartan users, 3/3 valsartan users, 2/2 losartan users, 1/1 eprosartan users). Seven cases (2.8%) reported recurrence of symptoms after receiving olmesartan. Rechallenge was not reported for the other ARBs.

### Comparative studies

Five comparative studies included in the presented study were retrospective analyses of large databases.

A case–control study by Marild *et al*. [26] linked histopathology-confirmed cases of villous atrophy to the Swedish Prescribed Drug Register. Each of the 2933 patients (median age, 28 years; 61.2% female) with villous atrophy recorded between July 2005 and January 2008 was matched to 5 controls and their ARB and ACEI use was examined. Olmesartan was not available in Sweden. Villous atrophy cases were less likely to have received a non-olmesartan ARB than controls, although this finding was not statistically significant (odds ratio [OR], 0.84; 95% confidence interval [CI], 0.64–1.09). There was no comparison of ARB vs. ACEI use directly; ACEI use was not associated with villous atrophy (OR, 1.08; 95% CI, 0.90–1.30). Among those with villous atrophy, 2.3% had used an ARB and 5.6% had used an ACEI prior to diagnosis compared with 2.7% and 5.2% use among the control population. Adjustments for age, sex and calendar year were performed.

A retrospective French study by Basson *et al*. [27] identified all adult patients initiating ARB or ACEI from January 2007 to December 2012 with a discharge diagnosis of intestinal malabsorption (ICD-10-CM codes, K90.x). A total of 860,894 person-years (PY) of olmesartan, 4,503,098 PY of other ARBs and 3,646,311 PY of ACEI exposure were reported. Mean age was 61.3 years for olmesartan, 62.3 years for other ARBs and 63.9 years for ACEI users. The ACEI users comprised fewer women (45.6%) than olmesartan (53.9%) and other ARBs (55.6%) users. The rate of hospitalization for intestinal malabsorption per 100,000 PY was 5.58 for olmesartan, 1.84 for other ARBs and 2.39 for ACEI users. This study showed an increased rate ratio of hospitalization due to intestinal malabsorption in olmesartan users (2.49; 95% CI, 1.73–3.57) and lower rate ratio of hospitalization due to malabsorption in other ARBs users (0.78; 95% CI, 0.58–1.07) compared to ACEIs users after adjusting for age, sex and the following comorbidities: heart failure, diabetes, immune-mediated diseases (rheumatoid arthritis, Hashimoto thyroiditis, IgA deficiency, dermatitis herpetiformis, lupus, Sjogren, dermatopolymyositis, complement deficiency, angioedema, inflammatory bowel diseases [IBDs]), active cancer and renal failure.

You *et al*. [28] conducted a retrospective study that included all Korean patients who were prescribed ARB or ACEI from January 2005 to December 2012 and were diagnosed and treated for intestinal malabsorption (KCD-6, K90.x based on ICD-10) in an inpatient or an outpatient setting. A total of 23,610 patients using olmesartan, 76,462 using other ARBs and 8487 using ACEI were reported. The mean age was 58.9 years for olmesartan (47.9% female) and other ARBs (48.4% female) users and 62.2 years for ACEI (45.4% female) users. The rate of enteropathy (<1 year) per 100,000 PY was 0.024 for olmesartan, 0.037 for other ARBs and 0.073 for ACEI users. The rate ratio of enteropathy among olmesartan (0.33; 95% CI, 0.10–1.09) and other ARB users (0.34; 95% CI, 0.14–0.83) was similar compared to ACEI users after adjusting for age, sex, income level, hypertension, dyslipidemia, heart failure, dementia, diabetes mellitus, autoimmune disease, chronic kidney disease, organ transplantation and cancer.

A German and Italian retrospective study conducted by Malfertheiner *et al*. [29] included all patients who initiated treatment with ARB and ACEI from 2004 to 2014 and reported hospitalization with a discharge diagnosis of intestinal malabsorption (Germany: ICD-10-CM codes, K90.x; Italy: ICD-9-CM codes, 579.x). A total of 32,035 PY of olmesartan, 136,818 PY of other ARBs and 431,123 PY of ACEI exposure were reported. Mean age was 63.0 years for olmesartan (49.7% female), 65.0 years for other ARBs (52.1% female) and 62.4 years for ACEI (46.3%) users. The rate of hospitalization for intestinal malabsorption per 100,000 PY was 12.49 for olmesartan, 14.62 for other ARBs and 9.05 for ACEI users. This study reported no significant differences in the rate ratio of hospitalization for intestinal malabsorption among other ARBs (1.06; 95% CI, 0.58–1.96) and ACEI (0.73; 95% CI, 0.37–1.46) users compared to olmesartan users after adjusting for age, sex and presence of at least one comorbidity among those of interest (diabetes, transplantation, malignant neoplasms and renal failure).

Five USA commercial claims and federal databases were pooled to compare olmesartan vs. other ARBs in this retrospective study conducted by Dong *et al*. [30]. This study included all patients who initiated treatment with an ARB between April 2002 and September 2015 and reported outpatient or inpatient visit with a diagnosis of celiac disease (ICD-9-CM codes, 579.0), malabsorption (ICD-9-CM codes, 579.8, 579.9), concomitant diagnoses of diarrhea (ICD-9-CM codes, 787.91, 564.5), weight loss (ICD-9-CM codes, 783.21) and non-infectious enteropathy (ICD-9-CM codes, 558.3, 558.9) were identified. A total of 350,790 eligible users of olmesartan and 1,577,679 users of other ARBs were reported. Mean age was 53.3 years for olmesartan (55.7% female) and other ARBs (55.7% female) users. The rate of inpatient visits for malabsorption per 1000 PY was 0.064 for olmesartan users and 0.054 for the users of other ARBs. This study reported an increased rate ratio of inpatient visits for malabsorption among olmesartan users as compared to other ARBs (1.25; 95% CI, 0.71–2.21) after adjusting for age, sex and presence of at least one comorbidity among those of interest (hypertension, ischemic heart disease, myocardial infarction, heart failure, cardiac arrhythmias, cerebrovascular disease, dyslipidemia, gastric bleeding, peptic ulcer, Crohn’s disease, osteoporosis, cancer, diabetes, transplantation and renal failure).

## Discussion

The present literature review included all case reports, case series and comparative studies reporting ARB-related enteropathy that were published before our search. We reported all cases reports and comparative studies published in the literature reporting ARB-related enteropathy. Our search identified 87 studies, of which 82 were case reports or series and 5 were retrospective studies. Overall, 248 cases of enteropathy associated with ARBs were reported with a fairly even male-to-female ratio and ages at diagnosis ranging from 45 to 89 years. Celiac disease-specific antibodies were negative in 99% of the cases. HLA DQ2 or DQ8 haplotype was present in 71% of patients [5, 6]. DQ2/DQ8 prevalence in the general population is estimated at 30%–40%, so genetics might play role in the onset of ARB-associated enteropathy [31]. ARB discontinuation resulted in clinical remission in 94% of cases. The majority of cases had used olmesartan.

The mechanism driving this adverse event is unclear. The most common causes of villous atrophy include celiac disease, medication, collagenous sprue and common variable immunodeficiency, human immunodeficiency virus, tropical sprue, giardiasis, whipple disease and viral disease [32]. ARBs may inhibit transforming growth factor, which maintains gut immune homeostasis [33]. The variable delay in onset of symptoms after ARB use suggests that it may be cell-mediated immunity damage rather than type 1 hypersensitivity, which typically shows a more immediate response [30]. Second, there are two types of angiotensin receptors—AT1 and AT2—that are expressed throughout the gastrointestinal tract [34]. A1 receptors maintain gut homeostasis and AT2 receptors induce epithelial cell apoptosis. Olmesartan was found to have more affinity for AT1 receptors and may saturate this receptor, allowing circulating angiotensin to bind unopposed to AT2 receptors, resulting in intestinal cell apoptosis and villous atrophy [34]. The affinity for Angiotensin receptors for other ARBs is not well understood. Villous atrophy was the most common histological finding and was reported in 90% of cases.

The comparative studies found that the absolute rate of enteropathy was low after the use of olmesartan, valsartan, irbesartan, losartan, telmisartan, candesartan, eprosartan and ACEI. Pooling of the comparative studies was difficult because of differences in the design. Studies had different periods of observation, different definitions of cases and different comparison groups. Studies that included cases diagnosed after May 2013 [5, 35] may have higher rates because of the awareness of the association between olmesartan and enteropathy. Case definitions included histologic confirmation from a pathology database, inpatient ICD codes, inpatient and outpatient ICD codes and different ICD codes to define the outcome. The comparison groups included olmesartan, other ARBs and ACEI users. Each of these design elements may have impacted the variation in the association between ARBs and enteropathy in the comparative study. The heterogeneity in comparison groups prevented pooling of results in a meta-analysis.

Our study has several strengths. We included cases using common criteria to define enteropathy across cases. The previous systematic reviews did not search for all ARBs [6, 10]. However, there were limitations. Not all studies reported the serology results. Therefore, we reported missing data. Second, the comparative studies were based on large databases conducted in different geographical regions with different case and period definitions.

## Conclusions

ARB-related enteropathy reported in the majority of case reports was associated with the treatment of olmesartan. In the 12 patients receiving other ARBs, discontinuation of the medication resulted in symptom remission. Olmesartan appears to be associated with a higher risk of enteropathy than other ARBs. ARB-related enteropathy should be considered a distinct clinical entity and should be included in differential diagnosis of diarrhea in hypertensive patients. Although enteropathy is rare, clinicians should remain vigilant when treating enteropathy, even after years following medication initiation.

## Authors’ contributions

A.K. acquired, analysed, interpreted data and drafted and critically revised the manuscript for important intellectual content. A.P. acquired and critically revised the manuscript for important intellectual content. C.F., E.G.V., D.L. and P.W. critically revised the manuscript for important intellectual content. S.H. conceived and designed this study, analysed and interpreted data, drafted and critically revised this manuscript, and supervised this study. All authors approved the final version of the manuscript.

## Funding

None.
